# Medico-Chirurgical Transactions; Published by the Royal Medical and Chirurgical Society of London

**Published:** 1842-07

**Authors:** 


### Art. IV.

*Medico-Chirurgical Transactions; published by the Royal Medical and Chirurgical Society of London. Second Series.* Vol. VI.—*London*, 1841. 8vo, pp. 258.

This upon the whole is an exceedingly creditable volume, containing some valuable information. We shall, as usual, give a pretty full abstract of its contents.

I. The first paper is a short but very interesting communication from Mr. G. Gulliver, "on some points connected with the anatomy of the entozoa belonging to the *genus cysticercus.*" The principal object of the communication is to show the situation and structure of certain oval corpuscules, of which the author had previously given an account in a paper read before the Zoological Society. If the white part near the head of the entozoon be gently pressed a little viscid fluid will escape, containing a great number of these bodies. Their figure is that of a short and generally somewhat flattened ellipse, the length being to the breadth in the proportion of rather less than 1½ to 1. Sometimes they are nearly circular. Their average long diameter is $\frac{1}{1352}$ English inch, their average short diameter $\frac{1}{2030}$ inch. They are of a whitish colour, smooth on the surface, and with a glistening appearance when seen by reflected light; viewed by transmitted light, they present a dark tinge towards the circumference, often with much brilliancy of surface, and a very clear and regular outline, but occasionally they have a dull white tint, and uneven margin. The majority are opaque, but in some a nucleus is visible; this is generally oval, but sometimes circular; it is most commonly situated in the centre of the corpuscule, but occasionally occupies a position near the edge. The texture of the nucleus is granular, as may be seen by compression.

The colour and opacity of the head and neck of the worm appear to be dependent upon the presence of these corpuscules, for the intervening tissue is nearly transparent. They are most numerous about the middle of the neck; their number diminishes towards the head, and they are but thinly scattered in the neighbourhood of the suctorious [?] orifices. They terminate suddenly where the neck expands into the bladder-like body of the worm. They are arranged in two layers, a superficial and a deep; the first appear ready to be cast off, for many of them will fall into a drop of water dabbed with [?] the part, and numbers can be removed by gently scraping the surface of the corium. They are soluble in muriatic and acetic acids, with evolution of gas. The solution gives a white precipitate with oxalic or sulphuric acid.

The corpuscules are not contained in any visible sac or cyst, but are situated in the parenchyma. Mr. Gulliver believes them to be the *ova* of the cysticercus, founding his opinion upon their heterogeneous structure, their regularity in size and shape, the aggregation in the true body of the worm, and the abundance of carbonate of lime contained in their shells. "I am not aware," he says, "that any gemmæ or sporules have yet been found to possess these characters; if, therefore, the oval corpuscules should be regarded as sporules, they must be sporules of a peculiar kind." We think these observations of Mr. Gulliver are well worth the attention of naturalists. It is the most commonly received opinion that the mode of generation of the cysticerci is gemmiparous, but in truth, the whole subject is as yet involved in no little obscurity, and much more extended observation will be necessary before definite conclusions can be arrived at. The ordinarily-admitted distinctions between ova and sporules are somewhat of an arbitrary character.

CHARITY HOSPITAL, MEDICAL LIBRARY.

The caudal vesicle contains a number of oil-like spherules, which Mr. Gulliver thinks are probably nuclei that advance to the neck of the worm, and there become invested with cells, and form complete oval corpuscules.

This paper also contains a good description of the hooks or spines which surround the proboscis of the worm.

Good drawings are given of all the parts described.

II. This is a very meager account of what is certainly (if there be no fallacy) a most remarkable "case of osseous union of a fracture of the neck of the femur," observed by Mr. Walter Jones, of Worcester. The subject was more than eighty years of age when the accident happened. After recovery the limb was shortened about one inch and a half, and considerably everted. The patient died two years afterwards. The neck of the bone was found much shortened and entirely osseous. We confess we should have liked greater details in the description. The nature of the case was probably satisfactory to those who had an opportunity of examining the specimens, but the readers of this communication will, we apprehend, find it anything but conclusive.

III. "Some observations on vaccination and small-pox," by Dr. G. Gregory. This is an interesting paper, and entitled to the fullest and most careful consideration.

The chief points adverted to are, the relation which subsists between the cicatrix and the character of the consecutive variola, and the theory of vaccine influence. We shall shortly notice both of these.

1. Three hundred and twenty-seven patients were admitted into the

Small-pox Hospital during the year 1840. Considerably more than half of these presented themselves in the course of the last three months. Eleven had complaints which proved not to be of a variolous nature. Of the remaining 316, 194 were wholly unprotected, and of these 87 died, or 45 per cent.; 120 had been previously vaccinated, and of these 8 died, being in the ratio of 7 per cent. only. Two were supposed to have previously undergone smallpox.

Dr. Gregory states that his attention had long been directed to ascertain whether any, and what relation subsisted between the number and character of the vaccine cicatrices, and the intensity of the consecutive variola; and he accordingly availed himself of the opportunity afforded by this epidemic for investigating the subject. The result was as follows: "In the majority of cases wherein the cicatrices are *numerous*, *normal*, and *well defined*, the consecutive variola is mild and varicelloid; again, where the consecutive disease proves severe, there the cicatrices will be imperfectly seen, or altogether wanting. But instances of the converse of these propositions are so numerous as scarcely to be called exceptions to a rule." In illustration of this remark, two series of cases are given, in the first of which smallpox was *severe* when the cicatrices were normal; and in the second, the lightest and most truly varicelloid eruptions coexisted with *small* and *very imperfect* cicatrices. We recommend the perusal of these cases to our readers, as being most instructive, and we think confirmatory of the opinion now expressed by Dr. Gregory, and which we have always entertained, that "the character of the cicatrix depends more on the *accidental* or *secondary*, than it does on the *primary* or *specific* inflammation," and that consequently little reliance can be placed on it as a measure of vaccine protection. Attentive observation of the disparity in the topical effects of vaccination in different individuals, the result of various but obvious contingencies, had long ago brought us to this conclusion, which the effects of our own re-vaccinations, and especially those on the continent, only tended to confirm. For these reasons we have been prepared to expect that Dr. Gregory's acuteness and opportunities of observation would eventually lead him to alter his former estimate of the three kinds of cicatrices figured and described by him some years ago, and adopt that which we have just quoted.

2. On the theory of vaccine influence. Dr. Gregory is not a believer in the identity of the variolous and vaccine poisons. He enumerates five distinct methods by which vaccinia may be produced: 1, By *spontaneous* generation in the cow; 2, by *contagion;* 3, by inoculation with the matter of *grease;* 4, by what is called *retro-vaccination;* 5, by inoculation with the matter of *human smallpox*, as clearly shown by the valuable experiments of Mr. Ceely, of Aylesbury, already fully noticed in this journal. (Brit. and For. Med. Rev., X., p. 464.) Upon these he remarks as follows:

"When we consider that five modes of producing this morbid secretion in the cow are now known to exist, it is not unreasonable to suppose that others may hereafter be discovered. In this state of our knowledge, then, surely we cannot be justified in assuming the fifth, and the last discovered of the whole, as the most important, and as affording the true clue to the mystery of vaccine protection. We should reflect that Mr. Ceely's experiments have entirely set aside Dr. Jenner's notion that vaccinia was the original or primitive poison, which time and fortuitous circumstances had aggravated into the malignant or secondary form, which we call smallpox. They have proved (if indeed they have *any* bearing on the intimate nature of these poisons) that smallpox is the *primary*, and cowpox the secondary form. But when we further reflect on the absence of a contagious principle in vaccinia, and the remarkable fact that febrile disturbance is not essential to its perfect development, we shall probably be nearer the truth in saying that the vaccine is a poison *sui generis*; that its relation to variola is still hypothetical; that the real and intimate nature of the protection which it affords is still unknown to us; and that a thorough acquaintance with its anti-variolous powers must be derived, not from analogy, but from an extended and careful observation of facts, through a long series of years." (pp. 27-8)

We confess these arguments do not carry great weight with us. The origin of vaccinia from inoculation with the matter of *grease* is more than doubtful; and if it be the case, which is most probable, that the horse is liable to a varioloid affection resembling that of the cow (vide Brit. and For. Rev., IX. p. 78), and capable of producing the true vaccine vesicle in that animal, *as well as in man*, the question is evidently much simplified, for the poison may certainly, without any undue stretch of hypothesis, be regarded as identical in its nature in all these instances. Again, it should be remembered that the subject of the spontaneous development of the disease in the cow has scarcely yet been examined with sufficient care, we mean in its connexion with the existence or non-existence of smallpox in the same locality. In the present state of our knowledge, we cannot help thinking that Mr. Ceely's experiments go *very far* towards proving the identity of the two poisons; it seems much more probable that the inoculation of smallpox matter into the cow should produce a fluid of the same, though a modified, nature, than that it should originate a poison totally *sui generis*. At the same time we quite agree with Dr. Gregory that years of extended and careful observation may be required before we shall be enabled to form any definite and satisfactory conclusions.

IV. "On Gouty Concretions." By Dr. Alex. Ure. The object of this paper is to introduce to the notice of the profession the *benzoic acid*, as a remedy calculated to prevent or remove these morbid depositions. The following is the rationale of the proposed method of treatment.

The tophaceous concretions, or chalk-stones as they are called, consist of urate of soda, with occasionally a small proportion of urate of lime. One part of urate of soda requires about 4000 parts of water to dissolve it, and this is probably the cause of the refractory nature of the above deposits. But the *hippurate of soda*, which exists in the urine of graminivorous animals, as the horse and cow, is an exceedingly soluble salt, requiring only two parts of water at 60° Fah. to dissolve one. It therefore occurred to Dr. Ure, that if by any means the hippuric could be substituted for the uric acid in the secretion of the human kidney, much good might result. Experiment showed that this could be readily effected.

"If, an hour after a meal, a scruple of benzoic acid be taken into the stomach, in the course of a couple of hours subsequently the urine voided, amounting to five or six ounces, will be found on adding a small quantity of muriatic acid, to yield a copious precipitate of beautiful rose-pink acicular crystals, which weigh, after being allowed to settle for a day, about fifteen grains. This quantity is by atomic computation equivalent to little more than half the benzoic acid employed."

Nearly the same results are obtained by employing the benzoate of ammonia or potass. No traces of uric acid or its salts, nor of benzoic acid can be detected in the urine after this treatment. No affection of the general health, nor irritation of the urinary organs was observed during the course of these experiments, which Dr. Ure first tried upon himself, afterwards upon individuals labouring under gout. Dr. Ure deserves great credit for being the first to suggest this philosophical plan of treatment, which we recommend to the earnest attention of our readers.

V. A "case of Phlebitis," by T. H. Silvester, M.D. The most remarkable features in this case were, 1, the situation of the disease, in the veins of the upper lip, side of the nose, and scalp; 2, its insidious progress, commencing from the irritation of a pimple, with the mild symptoms of a common cold, without violent shivering or delirium, the pulse and countenance alone betraying the severe nature of the affection, and these even regained their ordinary tranquillity in the course of the affection, while the patient's appetite never failed; 3, the appearance of a peculiar exudation along the course of the veins, which formed scabs, resembling those of *rupia;* when these were artificially removed, purulent matter escaped, and an ulcerated opening of the vein was found; but if they were allowed to fall off spontaneously, the vessel underneath was seen to be filled up with lymph. The patient was emaciated to the highest degree at the time of his death.

Why does Dr. Silvester use the French word "foyer," for a collection of purulent matter? Surely his own language could have furnished him with a term, at least as expressive and correct.

VI. Mr. Cæsar Hawkins has related "four cases of cancerous or malignant disease of the spinal column." We pass over the first, in which the patient died from mechanical obstruction of the bowels, by the pressure of two ovarian tumours upon the rectum, while the local disease was yet in an early stage; and proceed to give some account of the second, which presents also several interesting physiological features.

Jane Hall, æt. 55, was admitted for paraplegia. Her right breast had been removed six years previously for cancer, and she had remained well until May, 1839, when some cancerous tubercles formed in and around the cicatrix. Two months before the appearance of these tubercles, she began to complain of pain in the dorsal region of the back, and this was gradually followed by the ordinary symptoms of paralysis. The only appearance deserving notice in the back, was the projection of the spinous process of the sixth dorsal vertebra, which formed an acute angle with the rest of the column. Pressure upon this point, or anywhere below it, and for a little way above, gave rise to much increase of the pain.

Below the affected part of the spine the functions of the various organs were materially impaired, the patient, when attempting to sit up, feeling "as if she was about to drop into two parts." 1. All *voluntary muscular power* was totally lost, except *occasionally* over the expulsor fibres of the rectum. Many of the paralytic muscles were in an almost constant state of tonic spasm, and there were, at times, convulsive or clonic, and *painful* spasms in the same, or other muscles of the limbs and abdomen. The feet and legs did not much participate in this tonic spasm, nor in the occasional convulsions, and their muscles did not act nearly so much from external impressions, such as pressure, or pinching any part of the limbs, or tickling the soles of the feet, which readily excited the action of the muscles of the hip and knees. The sphincters of the anus and bladder were permanently paralysed. The bladder still retained its expulsive power, so that there was never any partial retention, but, unlike the rectum, it was entirely removed from the dominion of the will. 2. The *sensibility to external impressions* was entirely lost in every part below the seat of the disease, so that she could not feel smart pinching or pricking, or heat or cold in any part. The outlets of the bladder and rectum were also utterly insensible. Yet the spasms, whether spontaneos or excited, caused violent pain; and there was occasionally severe pain in the limbs even in the absence of all spasm. She had also much suffering in the abdomen, but this was probably dependent upon the cancerous disease which was detected after death in the peritoneum. 3. The *temperature* of every part below the disease was permanently exalted, being about 4° higher than that of the upper half of the body, allowing for difference in clothing, &c. 4. The secretions of the kidneys and intestines were, as usual unhealthy. The urine was alkaline, but contained no mucus. 5. The circulation and nutrition of the paralytic parts were impaired in the common way, sloughs and bullæ forming. The mortification on the hips and nates was the immediate cause of death.

On examination, all the vertebræ were found unusually soft and vascular, and in several, spots or tubercles of a yellowish white substance were found in the lateral plates. The medulla presented no appearance of inflammation. Opposite the sixth dorsal vertebra, it had been pressed upon by a tumour projecting from the body of the bone, and a deep sulcus of the entire circle formed, to the extent of fully one inch and a half; so little medullary matter remained in this portion, that the centre was almost transparent. The morbid structure which formed the tumour occupied three of the vertebræ, the sixth or central one being the most altered in shape. Four oval prominences projected into the canal from the body of this bone, over one of which an oval opening of the dura mater with smooth edges had been formed, apparently by absorption rather than ulceration. The new growth was of firm consistence, composed of fibrous structure of a white appearance in bands, with some yellow softer matter in the interstices. The other morbid changes discovered need not detain us.

This is certainly a very interesting case; not only as presenting an example of a rare form of disease, scirrhus of bones being much less common than the fungous and medullary varieties, but from the physiological symptoms to which the pressure upon the cord gave rise. It is evident from the facts stated above, that the muscular irritability of the paralytic limbs was *augmented* rather than impaired. The excito-motory phenomena were also well marked, and yet there was total loss of sensation: this is another addition to the now constantly accumulating proofs of the correctness of Dr. Marshall Hall's views on this point.

But the narrative of this disease brings before our notice some other features, of which it is extremely difficult to find a satisfactory explanation. How for instance, shall we account for the occasional voluntary power over the detrusor muscles of the rectum? and for the entire absence of any such control over the bladder, although its coats were equally free from palsy? What reason can be stated for the permanently increased temperature of the affected parts? Dr. Abercrombie explains phenomena of this nature by their having lost in some degree the power of preserving a medium temperature, and being consequently more affected by external circumstances; but Mr. Hawkins informs us, that, even when allowance was made for such influences, there was still a difference of 4° between their temperature, and that of the healthy parts.

We must advert to one other symptom, namely, the *spasms*. These have been commonly regarded as pathognomonic of disease of the membranes; but in the case before us, no such cause existed, for the opening through the dura mater was, according to Mr. Hawkins's account, the effect of absorption alone. We have ourselves met with at least two cases in which precisely the converse of this obtained, i. e. extensive disease of the spinal membranes, and no spasms; and we are, therefore, greatly inclined to think that the ordinary opinion is not so well founded as has been imagined.

In the cases above noticed, the disease of the spine was secondary to cancer of the breast; but the paper before us contains the record of two others, in which it was the primary affection. We have not space for them here, and must content ourselves with the simple statement, that one of them is regarded by Mr. Hawkins as an example of scirrhus, (though the description of the morbid structure does not appear to us very satisfactory,) while the other is an instance of fungous disease in a child, and affords another lamentable example of the little success which too often attends operations for the removal of these growths from the bones of the face. The whole paper is well deserving of an attentive perusal.

VII. Mr. Halberton has related a "case of slow pulse with fainting fits," which is chiefly remarkable for the very gradual development of the symptoms, and their apparent connexion with an injury of the neck received two years previously. We say *apparent* connexion, because the history of the case is somewhat defective, and there were also changes in the structure of the heart which throw some doubt upon the alleged origin of the affection.

VIII. The next article is a fourth memoir by Dr. Marshall Hall, "on some of the principles of pathology of the nervous system," and is equally interesting and important with its predecessors. It therefore claims from us a fuller notice than most of the papers with which it is associated. In this memoir, Dr. Hall directs attention to a very important topic, without an acquaintance with which, no scientific principles, however elevated their generality, however wide their scope, can be of any avail: this topic is, "How to observe." He had, on former occasions, remarked with justice, that almost all the diseases of the nervous system, or the groups of morbid phenomena which it exhibits, now require to be observed and recorded again, with express reference to his recent discoveries; and he now lays down the plan of observation which he considers most likely to produce valuable results. Of this plan and some of its applications we shall introduce a brief sketch, in the hope that the author's laudable design may be thereby in some degree furthered.

In considering the influence of any disease upon the nervous system, we must consider that system under three distinct divisions—the cerebral, the true spinal, and the ganglionic—and we must trace its influence upon each of these. This leads us to the following enquiries:—1. What *are* the distinct diseases of the cerebral, of the true spinal, and of the ganglionic subdivisions of the nervous system? 2. What is the influence of disease of one of these systems on the other two, respectively? 3. In what order is that influence manifested?

A disease which affects one portion of the nervous system exclusively, will present symptoms exclusively referrible to it. Thus, tetanus, in its simplest form, is exclusively spinal; whilst apoplexy or hydrocephalus is at first exclusively cerebral; and hemiplegia may be either cerebral, or cerebral and spinal. But it very commonly happens that diseases originally affecting only one portion of the nervous centres, extend their influence to others: this is the case, for example, with the severer forms of apoplexy and hydrocephalus which at first manifest themselves only in mental inactivity, more or less complete, but which subsequently affect the medulla oblongata, causing difficulty of respiration and deglutition, and may extend their depressing action to the whole spinal and ganglionic systems. "In cases of hemiplegia, the *danger* is precisely in proportion as spinal symptoms are superadded to those of the cerebral system. If the respiration be stertorous—if the deglutition be difficult—if the functions of the bladder, rectum, and sphincters be impaired, there is great danger; if these events *continue* for a considerable time, or if they *supervene*, the event is always fatal. . . . . . The spinal symptoms which exist at first and gradually yield, probably depend upon *counter-pressure* from congestion; this counter-pressure is relieved by blood-letting, &c., and its effects cease. When, on the contrary, the spinal symptoms continue, in spite of the remedies, they probably depend on the extent of the effusion; and this cannot be remedied." In the simple congestive apoplexy, also, the spinal system may become affected by the counter-pressure; and this is indicated by stertorous respiration, immobility of the eyelids, difficult deglutition. From this condition the patient may partially recover by means of free depletion; the breathing becoming more natural, a deep inspiration being excited by dashing cold water on the face, and the eyelids closing when the eyelashes are touched, without any decided advance towards consciousness—showing that the spinal symptoms are only consecutive upon the cerebral. If they *continue*, however, the issue must be fatal; for the patient will die of asphyxia. There is one remark of Dr. Hall's on this point with which we cannot accord. He says, "If the stupor and stertor continue, the next series of phenomena are those observed to result from defect of the functions of the ganglionic system. The bronchi become clogged with mucus, and the intestines distended or tympanitic from flatus." Now, Dr. J. Reid's experiments have clearly proved that the source of the effusion in the lungs noticed in cases in which the pneumogastric nerves have been divided, is to be found, not in any direct power of the nervous system over the secreting processes, but in the stagnation of the blood in the pulmonary vessels, which results from deficient respiratory action. We should not have noticed Dr. Hall's error (for such we believe it to be), were we not jealous of anything that can tend to perpetuate a doctrine which we believe to have had a most injurious influence on the progress of sound physiology—that of the immediate dependence of the functions of nutrition, secretion, &c., upon the ganglionic system of nerves. Tetanus is one of the most characteristic examples of a true spinal disease; the cerebral system being long spared, in fact, until a late period of the disease, when delirium or stupor manifests itself. In regard to the primary and secondary affections of the ganglionic system, our information is less precise; for the very obvious reason, that our knowledge of its normal functions is very far from being definite. "It is doubtful," Dr. Hall thinks, "whether any set of *diseases* originates in the ganglionic system; but there is a series of accidents which have their chief seat in this system. It is those which are caused by *shock*." That the nervous system has an influence, of which the nature is yet unknown, both upon the contractions of the heart and arteries, and upon the forces which move the blood in the capillaries, there can be, we think, but little doubt; and there can be as little, that this influence is conveyed by the ganglionic system. It may originate in any part of the nervous system; for concussion of the brain, sudden breaking down of the spinal cord, blows upon the epigastrium, or crushing a limb may produce the same effect. In the third of these cases, the impression is manifestly on the visceral nerves, which are derived from the sympathetic system; and this is probably true of the last also, since Dr. Marshall Hall has shown that the suspension of the circulation may be equally induced in a frog, by crushing its leg, after the spinal cord has been divided, as if it were uninjured. Dr. Hall thinks that, in exhaustion from loss of blood, we have a very good example of the successive affection of the cerebral, spinal, and ganglionic systems from the same cause. "The *cerebral* system is first affected; and then the previous symptoms of reaction give way to impaired vision and hearing, dozing and slight coma, and slight delirium when roused; then the *true spinal* system suffers, and the respiration loses its regular, even, and rhythmic character, and becomes slightly audible or stertorous, and each inspiration becomes accompanied with a sudden descent of the larynx—a symptom from which I have never known a patient recover; deglutition is slightly impaired, and the larynx is irritated to choking and violent coughing by the admission of fluids, whilst the sphincters of the rectum and bladder fail; lastly, the power of the ganglionic system fails too, and the respiration becomes marked by a slightly crepitous rattle, like the catching of the larynx—a fatal symptom—and the intestines become tympanitic. I have seen precisely the same order of symptoms, the same order of affections—first, of the cerebral, then of the true spinal, and lastly, of the ganglionic functions—from shock to the nervous system" by mental emotion.

Dr. Hall then directs attention to the principles of *irritation* and *pressure*, as causes of particular symptoms of nervous disorders. He remarks that, since it is a well-established physiological fact, that irritation of the cerebral substance itself cannot produce muscular movement, whenever spasmodic affections present themselves in nervous diseases, we must conclude that the spinal system is involved, either primarily or secondarily in the malady. Now, there are various modes in which cerebral disease may produce spasmodic actions through the spinal cord; as, for example, by counter-irritation or counter-pressure on the cord itself, or by involving its excitor nerves. Of the latter and least obvious mode of action a curious and important illustration is given in a postscript, in which Dr. Hall thus calls attention to the influence of irritation of the *membranes* of the brain in inducing *spasmodic* affections. "In an important experiment which I propose to lay before the Society in the next session, I found that, although every kind of irritation, puncture, laceration, &c., of the cerebrum and cerebellum was entirely inoperative, yet that laceration or pinching of the dura mater immediately induced peculiar spasmodic movements of the eyeball, the eyelids, the head, &c. These effects are probably induced through the branches of the trifacial nerve, which, as in the recurrent of Arnold, is well known to impart branches to the dura mater, and which may do so to other membranes within the cranium." It cannot be regarded as improbable that the surfaces of other membranes should be similarly furnished with excitor nerves, so that spasmodic diseases may result from states of irritation in them. This is a most fertile subject for experimental enquiry.

As, in all the secondary affections that have been alluded to, time is a most important element, we can no longer be surprised "that the same lesions, as found *post mortem*, have been attended by totally different series of symptoms during life, any more than that in the different periods of the same lesion, the symptoms have been different. . . . . It is not the *disease*, but its *effects* upon the brain and spinal cord, which are the source of the symptoms. If ramollissement, effusion, a tumour, &c., produce similar effects on these textures, the same affection of the functions, the same symptoms will be observed. . . . . . If the source of the symptoms be not the mere lesion of a function, induced by the lesion of a special part or organ of the encephalon, but the effect of irritation and counter-irritation, of pressure and counter-pressure, it is obvious that these primary effects, and their effects in their turn, may result from *any* disease, if the *times* be similar, whatever that may be. It is accordingly to the *history* that we chiefly have recourse for the diagnosis of cerebral diseases, and especially to those of the seizure and first stage: at their close, almost all diseases of the encephalon are alike; almost all terminate by coma, paralysis, convulsions, stertor, and impaired actions of ingestion and egestion of the orifices and sphincters, from compression of the cerebrum and medulla oblongata." Morbid changes take place towards the close of many diseases which do not properly or at all constitute the disease; on the other hand, the physical condition of some morbid states, such as that resulting from *shock*, *exhaustion*, &c., are of a kind which seem totally inscrutable, at least to our present means of research. The effects of cold in occasioning at first paralysis, and subsequently in many instances spasmodic tic, are also noticed by Dr. Hall as interesting subjects for enquiry. It is to the indication of such subjects, and of the mode of prosecuting the investigation, that the present memoir is devoted. We could wish that it had been more systematic in its form, especially in regard to its special object—"What to observe;" since we fear that those who have not made the subject one of especial study, will find it difficult to apply Dr. Hall's principles to the cases that may fall under their notice; and that a large amount of valuable data may thus be lost. The following table is given by Dr. Hall as a classified view of the points of enquiry to which he considers that our attention should be specially directed in cases of nervous disease.

i. The cerebral symptoms.
  1. Excess, or defect in the senses—pain.
  2. Delirium—coma.
  3. Paralysis.
ii. The true spinal symptoms.
  1. Spasm, clonic or tonic.
  2. Paralysis, in regard to
    *a.* The functions of ingestion.
    *b.* The functions of excretion.
    *c.* The muscular system generally.
iii. The ganglionic, in regard to
  1. Nutrition.
  2. Temperature.
  3. The secretions; especially those of
    *a.* The bronchi.
    *b.* The stomach and intestines.
    *c.* The kidneys and bladder.
iv. The effects of emotion.
v. The effects of shock.
vi. The effects of counter-pressure, &c.

We think it necessary to add, that, in recording the symptoms of a case of nervous disease under these heads, their *sequence* should be most carefully noted; the inferences from this being, as Dr. Hall has shown, frequently more important than those derived from the simple occurrence of the symptoms. We trust that his perseverance in following up the important enquiries to which he has devoted himself may be speedily rewarded by a splendid collection of such cases, "taken with the care and accuracy of M. Louis, and then as carefully analysed and compared."

IX. Mr. Stanley has given an account of "seven cases of spontaneous dislocation of the hip-joint." They cannot be satisfactorily abridged, and we have not room for their introduction in full; we must therefore content ourselves with simply directing the attention of our readers to them, with this passing remark, that Mr. Stanley appears to us to have passed over somewhat too slightly the local effects which may be rationally supposed to have resulted from the injuries mentioned in two, and the rheumatic affections which existed in three of the number. We certainly think it most probable that in most, if not all of these there must have been some low degree of inflammation, ending, perhaps, in effusion, and thus productive of elongation of the ligaments. It will be observed that in one case only (the second in which the dislocation occurred during paralysis from disease of the spinal cord) was there any dissection, and therefore the actual state of parts could only be supposed. The subject is one of considerable interest. We have no doubt that many surgeons have incurred most unmerited opprobium for the supposed fault of having overlooked dislocations, when in reality such were not in existence at the time of their examinations; having occurred subsequently, in a similar manner to those which Mr. Stanley has related, during the progress of convalescence or disease. The fact of such things being far from uncommon should be extensively known.

X. This is a short but interesting communication from Dr. Addison, "on some points in the anatomy of the lungs." The following is the result of his examination into the distribution and course of the *pulmonary veins.*

"The pulmonary artery was injected with size, coloured red, whilst the vein was injected with the same material, coloured yellow: the lung was then laid aside, and kept moistened in a cool place for several days, with a view of softening, by approaching decomposition, the connecting cellular membrane distributed throughout the lungs. In this way the common cellular membrane beneath the pleura became so lacerable that the pleura itself was stripped off without much difficulty, and without inflicting any breach whatever in the aeriel cellular structure of the lung which it had covered. The lung, thus divested of its pleura, presents to the eye, more or less distinctly, lines on its surface, which indicate the situation of what may be called the *pulmonary fissures* —a term more correctly applicable than that of interlobular, inasmuch as by the term interlobular is usually understood a something situated between either the longer lobes or smaller lobules; whereas, by the term *pulmonary fissures* is meant certain spaces occupied by common cellular membrane, and which descend from the surface towards the interior, but without penetrating the aeriel cellular tissue of the lungs; thereby dividing, more or less deeply, the surface of the organ into a number of insular portions, some of which may comprise a great number of lobules. Guided by the linear indications on the surface of the now naked lung, we can in general, with the aid of a pair of points, let into handles, or a pair of fine scissors, and without much difficulty, succeed in laying open and exposing the pulmonary fissures, at the bottom of which, merely surrounded by a loose cellular membrane, and resting on the unbroken aeriel pulmonary tissue, we discover a vessel; that vessel is the pulmonary vein—alone, and unaccompanied by any artery whatever. This vessel may be distinctly traced from larger to smaller trunks towards its source, until we reach the common cellular membrane between the ultimate lobules, from the exterior of which the vein appears to originate; whilst, on the other hand, by continuing the mechanical operation towards the root of the lungs, we with almost equal facility trace the vessel, still lying at the bottom of the pulmonary fissures,and becoming gradually larger and larger by the addition of branches, which proceed into the pulmonary fissure, and are derived either from the neighbouring smaller pulmonary fissures, or from the uniting cellular membrane between the ultimate lobules themselves, until at length it joins the large trunks at the root of the lungs to form the great pulmonary veins. A small artery is not unfrequently observed running across the pulmonary fissures, from a portion of lung on one side to a portion of lung on the other; and in one instance I have found an exceedingly narrow strip of healthy lung passing like a bridge across the fissure, on the very surface of the lung.

"Thus, then, the human lung may be said to be made up essentially of a vast expanse of membrane, the interior of which, during the whole of extra-uterine life, is unceasingly exposed to the influence of atmospheric air, and upon the surface or in the substance of which are spread out the capillary ramifications of the pulmonary artery; these arterial capillaries passing from thence to the exterior of the membrane to form the pulmonary vein, which throughout its whole course is found to be situated on the exterior of the aeriel cellular structure of the organs." (pp. 151-2.)

The application of these views to the purposes of pathological anatomy is sufficiently obvious. Dr. Addison thinks they will be the means of throwing additional light on the origin and progress of phthisis, and will enable us to set at rest the long-agitated questions respecting the origin and seat of pulmonary apoplexy, and more especially of what has been called *œdema pulmonium.*

XI. "A statistical account of the results of amputations at University College Hospital," by Mr. John Phillips Potter, late house-surgeon.

This is one of a class of papers which we hope to see continually multiplied. It is a lamentable fact, that little has been hitherto done in this country for the advancement of our science in the only path that can conduct to accurate and important generalizations; and we therefore hail, with the greatest satisfaction, every attempt, however humble, to fill up an hiatus that does so small credit to our philosophy.

The account before us extends over a period of six years and half, and comprises the results of 66 amputations on the shoulder, arm, forearm, wrist, thigh, and leg. The number is too limited to warrant the formation of any definite conclusions; but the report is valuable as far as it goes, and more especially because the cases were all treated on the same principles, and placed as nearly as possible under similar circumstances.

Of these 66 cases, 10 were primary amputations, (in the text the number is stated to have been 11, but the tables and calculations show that this must be an error of types,) and three of these proved fatal. Of the remaining 56, all of which were secondary operations, only 7 were lost. There were 22 cases of amputation of the thigh, with 4 deaths; 25 of the leg, with 3 deaths. Nearly all of the latter were performed close to the tuberosity of the tibia, leaving only a sufficient length of stump to fit firmly on the cushion of the wooden leg. The success attendant upon them is very different from that which has been procured in other hospitals. In the Glasgow Infirmary, for example, we find by the statements of Dr. Lawrie that fully one half proved fatal. (Lond. Med. Gaz. vol. i. p. 397.) How this discrepancy is to be explained, we cannot tell. The flap operation was adopted in all. With *very few exceptions* we believe this mode is decidedly preferable to the old circular method. The paper before us contains nothing new upon this point, beyond the advantage of forming the flaps of skin alone, when the leg is removed, immediately below the knee, and the patient is remarkably muscular. In only one case was the tourniquet applied, compression by the fingers being trusted to in all the others. There are many advantages in this plan, but the want of efficient assistance must often render it inapplicable in private practice. The paper closes by an account given by Mr. Liston of the mode of dressing which he employs. This eminent surgeon's views are now so generally known to the profession that we shall not occupy any more space with them here, than simply to bear our testimony to the striking success which we have often witnessed from their adoption. We should state also, that out of the whole number of cases there were only two instances of secondary hemorrhage, which is so much dreaded by the opponents of the flap method.

XII. "On the treatment of colica pictonum by warm water," by Dr. John Wilson. The mode advocated in this paper is the use of the warm bath, and the injection at the same time of some of the water into the intestinal canal. Copious evacuations have been procured in this way, with speedy relief of the pain, when all other remedies had failed. Cases illustrative of its success are narrated. The plan is applicable to other diseases in which obstinate constipation is a prominent symptom. It is very simple, and deserves trial.

XII. Dr. R. Boyd has related a case of "malposition of the kidneys, absence of the vagina, uterus, and fallopian tubes, and disease of the left ovary." The right kidney was found in the right iliac fossa; the left in the pelvis, below the psoas. The renal capsules were in their normal position.

XIV. "Pathological and surgical observations on diseases of the ear," by Joseph Toynbee, Esq., F.R.S. This is a very valuable paper, containing the first fruits of an enquiry conceived in a just spirit, and pursued with philosophical accuracy. It is perhaps less valuable, however, for what it does than for what it promises, and to which it will probably lead the way. It must be acknowledged that the science of surgery has hitherto thrown but a very faint light upon the nature of the diseases of the ear; indeed, this branch of surgery is so far in the rear of all others, that, in the *vast majority* of cases of deafness which fall under the observation of the surgeon, he is quite incapable of offering any diagnosis or of affording any relief. There is no truth-speaking member of the medical profession who has not made such a confession, and many who have tried the modes of relief which have been lately suggested, and found them inefficient, are inclined to believe that, as a general rule, deafness is an incurable disease. Under these circumstances, it becomes indeed most desirable that some light be thrown upon the true nature and the seat of this class of diseases; and, as is stated by the author of this paper, this end can be accomplished only by the aid of pathology.

Mr. Toynbee here points out the pathological changes which the structures composing the ear undergo, by giving the details of 41 dissections which he has conducted: 39 of the specimens were taken from patients who died of various diseases and at different ages, who during life were not known to be deaf. In pursuing this plan, with the expectation of discovering disease in its incipient state, he follows the example of Sir B. Brodie in his researches upon the Joints; and, like him, Mr. Toynbee finds disease in organs which were supposed to be healthy; and he is thus enabled to point out the seat and nature of the morbid changes at their origin. It appears that, of the 39 specimens examined, 29 presented well-marked pathological conditions in different stages. These may be divided into three classes: the *first* consisting of those in which the mucous membrane of the cavity of the tympanum is in a thickened state; the *second*, in which membranous bands connect various parts of the cavity of the tympanum; the *third*, in which the thickened state of the membrane is combined with the presence of the bands of adhesion. The appearances found in the healthy specimens are first described; from which it appears that the mucous membrane of the tympanic cavity presents the following characters when in a natural condition:

"The cavity of the tympanum contains but a very small quantity of mucus, which is spread over the surface of the investing membrane. The latter is so extremely thin and transparent, that its presence upon the surface of the osseous walls of the tympanum cannot be detected without the use of a magnifying glass, and by the aid of the touch. The nervous filaments upon the surface of the promontory are most distinctly seen; the margin of the fenestra rotunda is distinct and defined, and the membrane which closes it is thin and transparent. The ossicula at first sight do not appear to be covered by any membrane. The crura of the stapes and their point of attachment to its base are seen distinctly, and between the inferior surface of these crura and the promontory is seen a well-marked fissure." (p. 194.)

The author then proceeds to show that, in specimens wherein this membrane is thickened, it becomes opaque and white; the tympanic plexus of nerves is not discernible through it; the ossicles receive a thick investment from it; the stapes is completely concealed; the fenestra rotunda, instead of having the appearance of a defined foramen, presents a simple depression in the pulpy membrane: bands of adhesion most frequently coexist with this "soft and velvety" condition of the membrane, and they have been observed to connect the crura of the stapes to the membrana tympani and surrounding parts, and the malleus and incus to the surface of the promontory and to the membrana tympani; accompanying this diseased state of the membrane is an increased secretion of mucus.

The following is a concise view of the state of the cavity of the tympanum in the 41 dissections detailed in this paper:

| | |
|---|---|
| "1 In a healthy state | 10 |
| 2. With simple thickening of the investing membrane | 6 |
| 3. With bands of adhesion passing from various parts of the cavity of the tympanum, most frequently connecting the stapes to the circumference of the tympanic cavity | 4 |
| 4. With slight thickening of the investing membrane, accompanied by the existence of bands of adhesion | 13 |
| 5. With considerable thickening of the investing membrane and the presence of bands of adhesion | 5 |
| 6. Suppuration in the cavity of the tympanum | 1 |
| 7. Anchylosis of the base of the stapes to the circumference of the fenestra ovalis | 2 |
| (p. 208.) | 41" |

The author concludes his paper with observations to the following effect. He says, it must appear remarkable, that, out of 39 ears taken promiscuously, 29 should have presented appearances indicative of disease; he supposes, however, that in several specimens the deviation from the healthy state was so slight, that it may be presumed no derangement of the functions of the organ attended them. Nevertheless, he continues, the specimens in which the diseased conditions were considerable are so numerous as to cause surprise, which, however, will be diminished when it is considered that many persons who consider that they *hear perfectly well* cannot distinguish the ticking of a watch at a distance of two feet and a half, or even of four or five inches, which can be heard by the healthy ear seven or eight feet from the head. Dr. Wollaston wrote a paper in the Philosophical Transactions, showing that there was a very striking difference between the powers of hearing of different individuals; and as the defects to which Dr. Wollaston refers always occurred in persons more than twenty years of age, Mr. Toynbee is inclined to believe that they may be ascribed to the pathological conditions which he has pointed out.

XV. Mr. Roden has recorded "two cases of displacement of the long tendon of the biceps humeri." In the first it was unaccompanied with any other injury, and the tendon was found lying on the lesser tubercle of the humerus. In the second the bone had been dislocated forwards and upwards, and the tendon was found lying at the inner and posterior aspect of the joint, having completely slipped over the head of the humerus.

XVI. Dr. J. A. Wilson has given an account of "two cases of aneurism of the superior mesenteric artery," a rare form of disease. The first was particularly remarkable. The patient, a female, æt. twenty-four, suffered under jaundice of a very severe character, which proved fatal seven weeks after her admission. External examination of the epigastric region (where there was occasional pain) gave no indication of the existence of any tumour, and the true nature of the case was, of course, not suspected until after death—an excellent commentary upon the necessity of using the *ear* as well as the hand in all doubtful affections of the abdominal cavity. Upon dissection, it was found that the sac had compressed the ductus communis choledochus throughout its whole extent. In the second case the tumour was felt, and its structure recognized.

XVII. The last paper is one by Mr. Stanley, "on congenital tumours of the pelvis." The author considers that they may be arranged in four classes. 1. Those in which the tumours consist wholly of morbid tissues, generally a combination of the fibrous and cystic varieties. 2. Those in which they are composed of morbid structures, in combination with isolated portions of perfectly-formed animal organs, having no other relation to the living being with which they are connected, than as they are dependent upon it for the means of nutrition and growth. These belong to the class of parasitic monsters. 3. Spina bifida. 4. Those in which the tumour is composed—either wholly or in part—of membranous cysts, communicating with the spinal canal, but *exteriorly to the theca.*

Mr. Stanley mentions one fact which has considerable interest in a physiological point of view. In two of the cases examined an *isolated* portion of intestine was found in the parasitic monster, containing a fluid, "which in colour and other obvious characters closely resembled meconium, although there existed no liver or other distinct hepatic apparatus which could have furnished the colouring matter of the fluid, and there was certainly no communication between this portion of intestine and the intestinal canal of the child to which the parasitic monster was attached." We cannot help regretting that recourse was not had to chemical analysis; it would have been extremely important to have ascertained the presence or absence of other essential constituents of the bile besides the colouring matter.

A question naturally arises in reference to these tumours—should they be ever made the subjects of surgical operation? The paper before us shows that in some instances they might be removed with success; indeed Mr. Blizard has done so with the best results. But when we consider the almost absolute impossibility of determining the true nature of the case by external examinations, and the certainty of death if there were any communication with the theca of the cord, we feel greatly inclined to the opinion that it would be the part of a wise surgeon to decline all interference.

